# A Novel C-Terminal Truncated Bacteriocin Found by Comparison between *Leuconostoc mesenteroides* 406 and 213M0 Isolated from Mongolian Traditional Fermented Milk, Airag

**DOI:** 10.3390/microorganisms12091781

**Published:** 2024-08-28

**Authors:** Hasiqimuge, Chihiro Hano, Kensuke Arakawa, Saki Yoshida, Junliang Zhao, Hidehiro Toh, Hidetoshi Morita, Taku Miyamoto

**Affiliations:** 1 Graduate School of Environmental and Life Science, Okayama University, Okayama 7008530, Japan; 2 Department of Grassland Ecology, Animal Husbandry and Veterinary, Xilingol Vocational College, Xilinhot 026000, China; 3 Faculty of Agriculture, Kagawa University, Kagawa 7610795, Japan; 4 Advanced Genomics Center, National Institute of Genetics, Mishima 4118540, Japan; 5 Faculty of Food Culture, Kurashiki Sakuyo University, Okayama 7100292, Japan; 6 Microbial Fermentation Research Center, Minori Co., Ltd., Okayama 7011221, Japan; 7 Functional Food Creation Research Institute Co., Ltd., Okayama 7161241, Japan

**Keywords:** *Leuconostoc mesenteroides*, antimicrobial peptide, bacteriocin, *Listeria monocytogenes*, fermented milk, biopreservation, fermentation control, post-translational modification, C-terminal cleavage

## Abstract

Bacteriocins produced by lactic acid bacteria are known to be useful tools for food biopreservation and fermentation control. *Leuconostoc mesenteroides* subsp. *mesenteroides* 406 and 213M0 isolated from different samples of Mongolian traditional fermented milk, airag, had been reported to produce listericidal bacteriocin-like inhibitory substances with similar but slightly different properties. In this study, the antibacterial properties and the related gene sequences of both strains were compared, and then their bacteriocins were purified and identified. Strain 406 was superior to strain 213M0 in cell growth and antibacterial activity against many strains. However, the activity of 213M0 was stronger than that of 406 against a few strains. DNA sequencing revealed two and three plasmids in 406 and 213M0, respectively, and each one of them harbored an almost identical mesentericin Y105–B105 gene cluster. Removal of these plasmids resulted in a complete loss of activity, indicating that the antibacterial activity of both strains was generated by bacteriocins encoded on the plasmids. Mesentericins Y105 and B105 were purified from both cultures, and another novel bacteriocin, named mesentericin M, was identified from the 213M0 culture only. Its structural gene was coded on a 213M0 plasmid and, surprisingly, its C-terminal three amino acid residues were post-translationally cleaved. To our knowledge, this is the first report of a C-terminal truncated bacteriocin. In conclusion, the novel bacteriocin should be mainly responsible for the difference in antibacterial properties between the two strains.

## 1. Introduction

*Listeria monocytogenes* is recognized as one of the most important foodborne pathogens causing gastrointestinal diseases, septicemia, etc., leading to higher mortality in pregnant women, newborns, the elderly, and immunocompromised individuals [1]. To prevent contamination with *Lis. monocytogenes*, the use of antibiotics and chemical preservatives is generally effective in livestock and food production. However, in recent years, consumers are demanding more natural and minimally processed foods as their awareness of food safety increases, and they tend to avoid the risks associated with the use of antibiotics and chemical preservatives, such as disorders of their gastrointestinal microbiota and the development of antibiotic-resistant and preservative-tolerant bacteria [2]. In this context, bacteriocins produced by some strains of lactic acid bacteria (LAB) have gained attention in recent decades for their potential as natural alternatives to antibiotics and chemical preservatives [3,4]. Bacteriocins are ribosomally synthesized antimicrobial peptides or proteins that inhibit the other related (narrow-spectrum) or unrelated (broad-spectrum) bacteria.

The best known bacteriocin is nisin A, produced by some strains of *Lactococcus lactis* subsp. *lactis* [3,4]. Nisin A is the only bacteriocin approved by the U.S. Food and Drug Administration for use as a food preservative, and it is commercially available in more than 50 countries worldwide. Another commercially available bacteriocin is pediocin PA-1/AcH, produced by some strains of *Pediococcus acidilactici* and the related species LAB [3,4]. Nisin A and pediocin PA-1 are both excellent food preservatives due to their strong and broad-spectrum antibacterial activity against food spoilage bacteria and foodborne pathogens, including *Lis. monocytogenes*. On the other hand, due to their broad spectra, they are considered unsuitable as fermentation controllers that require beneficial LAB to be kept alive [4]. In this aspect, bacteriocins with narrow spectra that inhibit only certain spoilage and pathogenic bacteria such as *Lis. monocytogenes* are desired as food fermentation controllers.

*Leuconostoc* spp. are LAB familiar to humans, frequently detected in traditional fermented foods, and used industrially as starter bacteria for food fermentation [5]. Some strains of *Leuconostoc* spp. produce bacteriocins, and a variety of *Leuconostoc* bacteriocins have been reported in the last 40 years since the first discovery in 1984 [5,6,7,8,9,10,11,12,13,14,15,16]. Most of them have antibacterial activity, especially against *Lis. monocytogenes* [5,8,9,10,11,13,14,15,16], and some strains produce more than one bacteriocin. For example, *Leu. mesenteroides* TA33a, isolated from spoiled meat, produces three bacteriocins, leucocin A, B, and C [14], and *Leu. pseudomesenteroides* QU 15 produces leucocin A, Q, and N [15]. Furthermore, *Leu. pseudomesenteroides* 607, isolated from persimmon fruit, produces leucocin C-607 together with another unknown bacteriocin [16].

*Leu. mesenteroides* subsp. *mesenteroides* 406 and 213M0 were isolated from different samples of Mongolian traditional fermented milk, airag, in our laboratory [17,18]. The cell-free culture supernatant (CFS) of the two strains showed narrow antibacterial spectra, especially with no activity against lactobacilli, but was highly effective against *Lis. monocytogenes*. The activity was stable under broad pH range and against heat treatment, but labile to proteases. These results suggested that both strains probably produced narrow-spectrum listericidal class IIa bacteriocins with promise as fermentation controllers. In addition, draft genome sequences [19,20] showed that the two strains had a gene cluster responsible for biosynthesis and immunity for already known narrow-spectrum listericidal bacteriocins, mesentericins Y105 and B105 [21,22,23,24,25,26]. This correspondence of the gene clusters was a bit strange, because the size of the bacteriocin-like inhibitory substance (BLIS) produced by strain 213M0 was thought to be slightly larger than that of strain 406, based on the result of an in situ antilisterial activity assay [18]. Therefore, more comparative analysis would be needed, as the two strains were previously studied separately under different conditions.

Toward the ultimate goal of using *Leu. mesenteroides* subsp. *mesenteroides* 406 and 213M0 as food biopreservatives and fermentation controllers, the aim of this study was to clarify the differences between their bacteriocins by recomparing the antibacterial properties and bacteriocin-related gene sequences and by purifying and identifying their bacteriocins.

## 2. Materials and Methods

### 2.1. Bacterial Strains and Culture Conditions

*Leu. mesenteroides* subsp. *mesenteroides* 406 and 213M0 were cultivated with 2% (*v*/*v*) inoculum in MRS broth (Oxoid Limited; Hampshire, UK) at 25 °C. Other strains used in this study are listed in Table 1 along with their culture conditions. Tryptone, yeast extract, lactose, and glucose (TYLG) broth [27] was used to propagate *Listeria* spp. strains. The ingredients of TYLG broth were purchased from Becton, Dickinson and Company (Franklin Lakes, NJ, USA) and FUJIFILM Wako Pure Chemical Corporation (Osaka, Japan). Before use, all bacterial strains were propagated three times.

### 2.2. Antibacterial Activity Assay

The agar well diffusion method [28] was used to determine antimicrobial activity. To prepare CFS as a sample for the assay, the 24 h incubation culture of both strains was neutralized (pH 7.0) with 1 M NaOH, followed by centrifugation at 1600× *g* for 20 min at room temperature, and sterilized through a 0.22 μm filter (Membrane Solutions, LLC; Auburn, WA, USA). CFS was then serially diluted two-fold using sterile saline. 

Each indicator strain listed in Table 1 was inoculated at 2.5% (*v*/*v*) into an appropriate agar medium and mixed well. After solidification, wells (6 mm diameter) were punched on the agar plate. CFS or the dilution (50 μL) was aliquoted into the well, and the plate was incubated at 30 or 37 °C for 24 h. 

A clear zone without cell growth of each indicator around the well indicated the presence of BLIS or bacteriocins. The unit of BLIS/bacteriocin activity (arbitrary units, AUs) was defined as the reciprocal of the highest dilution inhibiting the growth of each indicator strain. Results presented are the mean ± S.D. of at least three independent experiments.

### 2.3. Sequential Measurement of Bacterial Growth and Bacteriocin Production

Bacterial growth of strains 406 and 213M0 was evaluated by sequentially measuring culture pH, turbidity (at OD_620_), and viable cell counts at 0, 8, 16, 24, 48, and 72 h of incubation. At the same time, bacteriocin production was also evaluated by measuring the antibacterial activity of CFS against *Lis. monocytogenes* VTU 206 as an indicator.

### 2.4. Antibacterial Spectral Assay

Antibacterial spectra of strains 406 and 213M0 were evaluated by the antibacterial activity assay of CFS against 32 bacterial strains listed in Table 1.

### 2.5. PCR and DNA Sequencing to Close Plasmid Gaps

In previously reported draft genome information of strains 406 (BCMP01000000) and 213M0 (BCMO01000000) [19,20], two and three plasmid-like sequences are shown as contigs-33 and -40 in 406, and contigs-26, -30, and -48 in 213M0. The gaps in these contigs were filled by PCR and DNA sequencing to obtain the full-length circular plasmid sequences.

Total DNA and plasmid DNA from both strains were extracted and purified as described by Klaenhammer (1993) [29].

PCR was performed with primers (Table 2) designed based on the contig sequences and KAPA Taq EXtra HotStart ReadyMix (Kapa Biosystems, Inc.; Wilmington, MA, USA) using SimpliAmp Thermal Cycler (Applied Biosystems; Waltham, MA, USA). The PCR conditions for contigs-33 and -40 in strain 406 and contigs-30 and -48 in strain 213M0 were as follows: initial heating at 95 °C for 2 min; 40 cycles of 95 °C for 30 s, 58 °C for 30 s, and 72 °C for 30 s; and final extension at 72 °C for 7 min. For contig-26 in 213M0, first, primers 213-26-F5 and -R5 were used under the following conditions: initial heating at 95 °C for 2 min; 40 cycles of 95 °C for 30 s, 52 °C for 30 s, and 72 °C for 30 s; and final extension at 72 °C for 7 min. Next, the PCR amplicon was extracted using MagExtractor DNA Fragment Purification Kit (Toyobo Co., Ltd.; Osaka, Japan) after agarose gel electrophoresis, and then used as a template for the second PCR with primers 213-26-F4 and -R4 under the following conditions: initial heating at 95 °C for 1 min; 40 cycles of 95 °C for 30 s, 60 °C for 1 min, and 72 °C for 30 s; and final extension at 72 °C for 7 min.

The resulting PCR amplicons were extracted after electrophoresis and finally submitted to DNA sequencing service at Eurofins Genomics K.K. (Tokyo, Japan). DNA sequences obtained were submitted to the DNA Data Bank Japan (DDBJ) to obtain accession numbers (LC832857-LC832861) after annotation using the DDBJ Fast Annotation and Submission Tool (DFAST). In addition, the putative genes related to bacteriocin biosynthesis and immunity were analyzed using the Basic Local Alignment Search Tool (BLAST) of National Center for Biotechnology Information (NCBI).

### 2.6. Plasmid Curing

Strains 406 and 213M0 were cultivated stepwise in the modified MRS broth supplemented with novobiocin (1, 2, 5, 10, 20, 50, 100, to 200 μg/mL) to obtain derivative strains. The loss of plasmids pLM406A (contig-33 of strain 406) and pLM213M0A (contig-26 of strain 213M0) in the derivative strains was confirmed by PCR using primers of mesY-F and -R (Table 2) to detect the structural gene (*mesY*) of mesentericin Y105. Primer pairs of 406-40-F1 and -R1, 213-30-F1 and -R1, and 213-48-F2 and -R2 (Table 2) were also used to detect plasmids pLM406B (contig-40 of strain 406), pLM213M0B (contig-30 of strain 213M0), and pLM213M0C (contig-48 of strain 213M0), respectively. PCR was performed using KAPA2G Fast HotStart ReadyMix (Kapa Biosystems). The PCR conditions were as follows: initial heating at 95 °C for 10 min; 35 cycles of 95 °C for 15 s, 53 °C for 15 s, and 72 °C for 5 s; and final extension at 72 °C for 1 min. Only for pLM406B, KOD One PCR Master Mix (Toyobo) was used, and the PCR conditions were as follows: initial heating at 98 °C for 10 min; 40 cycles of 98 °C for 10 s, 53 °C for 5 s, and 68 °C for 5 s; and final extension at 72 °C for 30 s. After PCR, agarose gel electrophoresis was performed with FastGene 50-bp DNA Ladder (Nippon Genetics Co., Tokyo, Japan) to visualize the loss of each plasmid.

The antibacterial activity of the derivative strains with loss of plasmids was tested against seven indicator strains: *Lis. monocytogenes* VTU 206, *W. paramesenteroides* JCM 9890^T^, *Leu. lactis* JCM 6123^T^, *E. faecalis* JCM 5803^T^, *Leu. mesenteroides* subsp. *dextranicum* NBRC 100495^T^, *W. cibaria* JCM 12495^T^, and *Leu. mesenteroides* subsp. *cremoris* NBRC 107766^T^.

### 2.7. Bacteriocin Purification

Purification of bacteriocins was performed according to Biet et al. (1998) [24] with some modifications. First, each CFS (100 mL) prepared by centrifugation (23,800× *g*, 20 min, 4 °C) of culture solutions of strains 406 and 213M0 was heated at 70 °C for 30 min. Next, ammonium sulfate (35%, *w*/*v*) was added to the heated CFS, and the mixture was stirred at 4 °C for 18 h to precipitate the bacteriocins. The precipitate was collected by centrifugation (5900× *g*, 1 h, 4 °C) and redissolved in 10 mL of distilled water. The solution was then applied to a reverse-phase Sep-Pak C18 cartridge (Waters Corporation; Milford, MA, USA). Elution was performed with 5 mL of 0, 15, 30, and 40% (*v*/*v*) acetonitrile in 20 mM ammonium acetate. All eluates were concentrated to 200 μL using a centrifugal evaporator (model EC-57C, Sakuma; Tokyo, Japan). The concentrated eluate with antibacterial activity was further subjected to reverse-phase HPLC (Shimadzu Corporation, Kyoto, Japan) with a Wakosil-II 5C8 RS HPLC column (4.6 mm × 250 mm; FUJIFILM Wako Pure Chemical Corporation) equilibrated with solvent A (10% acetonitrile and 0.1% trifluoroacetic acid, *v*/*v*) at 50 °C. Elution was performed at a flow rate of 1 mL/min with a linear gradient from 0 to 100% of solvent B (90% acetonitrile and 0.1% trifluoroacetic acid; *v*/*v*) for 45 min. The eluate was monitored at a wavelength of 220 nm, and the eluate peaks shown were collected. After that, the collected fractions were concentrated and assayed for antibacterial activity against three indicator strains: *Lis. monocytogenes* VTU 206, *W. paramesenteroides* JCM 9890^T^, *Leu. lactis* JCM 6123^T^.

Peptide content of the collected samples at each purification step was determined using Pierce Micro BCA Protein Assay Kit (Thermo Fisher Scientific Inc.; Waltham, MA, USA). 

### 2.8. N-Terminal Amino Acid Sequencing

The bacteriocins purified by HPLC were denatured at 130 °C for 30 min, and dissolved in 40% (*v*/*v*) acetonitrile. The solution was loaded several times onto a PVDF membrane hydrated with methanol and distilled water and then completely dried at 55 °C. After that, the membrane was applied to N-terminal amino acid sequencing using a PPSQ-31A peptide sequencer (Shimadzu Corporation) at the Department of Instrumental Analysis in Okayama University, Japan.

### 2.9. Mass Spectrometry Analysis

Dried samples after purification by HPLC were dissolved in 10 µL of 70% acetonitrile (*v*/*v*) containing 0.1% (*v*/*v*) formic acid and mixed with a saturated solution of α-Cyano-4-hydroxycinnamic acid used as a matrix at a volume ratio of 1:5. The mixture was applied to MALDI-TOF MS or MS/MS analysis using UltrafleXtreme (Bruker, Billerica, MA, USA) at the Department of Genomics and Proteomics in Okayama University, Japan. The data obtained from MS/MS were further analyzed to determine the peptide sequence using the Mascot server.

## 3. Results

### 3.1. Comparison of Bacterial Growth and Bacteriocin Production

**Figure 1:**
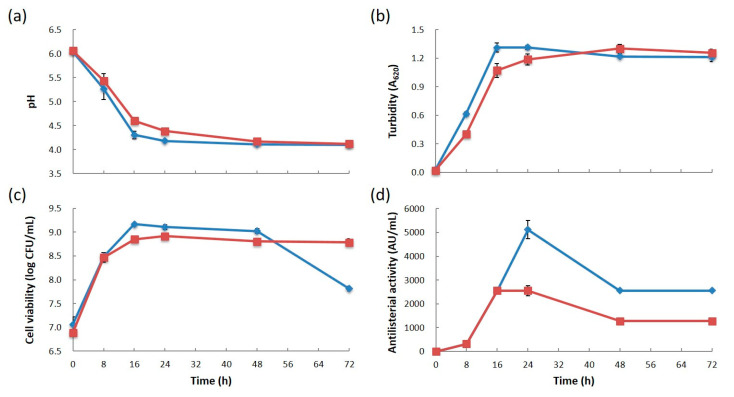
Changes in culture pH (**a**), turbidity (**b**), cell viability (**c**), and antilisterial activity against strain VTU 206 (**d**) of *Leuconostoc mesenteroides* subsp. *mesenteroides* 406 and 213M0. Blue and red indicate strains 406 and 213M0, respectively.

The culture pH, turbidity, and cell viability in the MRS broth were measured sequentially for 72 h to compare the growth of *Leu. mesenteroides* subsp. *mesenteroides* 406 and 213M0. Strain 406 showed significantly better growth than 213M0 except for the decline phase (Figure 1a–c). At 16 h incubation, the cell viability of strain 406 (1.47 × 10^9^ CFU/mL, pH 4.3) was more than twice that of strain 213M0 (7.08 × 10^8^ CFU/mL, pH 4.6).

The antilisterial activity of their CFSs against strain VTU 206 was also measured sequentially for 72 h to compare the bacteriocin production of strains 406 and 213M0. The activity of both strains increased in parallel with the cell growth during the exponential phase up to 16 h of incubation (Figure 1d). After 16 h of incubation, the activity of strain 406 was twice as high as that of 213M0. In addition, from the end of the stationary phase to the decline phase during 48–72 h of incubation, the activity of both strains was reduced by half compared with the maximum activity at 24 h of incubation.

These results suggested that the differences in antibacterial properties of both strains might be due to differences in bacteriocin production affected by cell growth.

### 3.2. Comparison of Antibacterial Spectra

The antibacterial spectra of strains 406 and 213M0 were compared against 32 bacterial strains, including *Listeria* spp. and LAB. Both CFSs showed similar antibacterial spectra (Table 1), in that the CFS inhibited the growth of all tested strains of *Listeria* spp. and some strains of *Enterococcus*, *Leuconostoc,* and *Weissella* spp. but had no activity against all tested strains of *Lactococcus, Pediococcus,* and *Streptococcus* spp. In addition, the activity of strain 406 was generally higher than that of strain 213M0. However, only against two strains, *Lis. monocytogenes* JCM 7680 and *Leu. lactis* JCM 6123^T^, the activity of both strains was reversed; namely, the activity of 213M0 was higher than that of 406. These results suggested that there would be differences not only in bacteriocin production, as noted above, but also in the antibacterial substances themselves.

### 3.3. DNA Sequencing and Plasmid Mapping

Plasmid-like contigs (contigs-33 and -40 in strain 406 and contigs-26, -30, and -48 in strain 213M0) in the previously published draft genome sequences [19,20] were gap-filled by PCR and DNA sequencing. Among them, contig-33 in 406 and contig-48 in 213M0 were cyclized without any sequence insertion. Contig-40 in 406 and contig-30 in 213M0 were directly cyclized with 248- and 3-nt sequences, respectively. The other one, contig-26, included a 171-nt wrong sequence at the 5′-terminal side. In addition, another 134-nt sequence (at the 5′-side of contig-54) was added to its 3′-end to be cyclized as a plasmid. As a result, two (named pLM406A and pLM406B) and three (named pLM213M0A, pLM213M0B, and pLM213M0C) plasmids were confirmed in strains 406 and 213M0. 

The sequences of the five plasmids were annotated using DFAST. Table 3 and Supplementary Figure S1 show an overview and maps of the sequenced and annotated plasmids. Both pLM406A and pLM213M0A contained the mesentericin Y105-B105 gene cluster (Supplementary Figure S1a,c), and pLM213M0B encoded two genes (*mesDE*) related to a transport system of mesentericins (Supplementary Figure S1d). In addition, pLM406B contained genes related to a citrate metabolism system (Supplementary Figure S1b), and pLM213M0C was a cryptic plasmid (Supplementary Figure S1e). Incidentally, no gene sequences thought to be related to bacteriocins were found in the chromosome contigs.

### 3.4. Homology Analysis of Mesentericin Y105–B105-Related Genes

**Figure 2:**
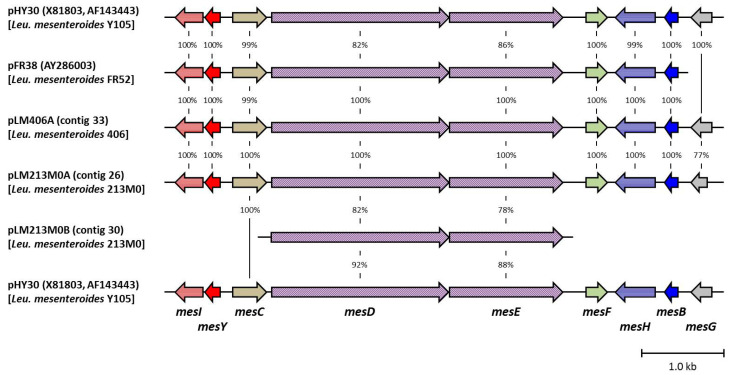
Sequence alignment of mesentericin Y105–B105-reated genes. Gene functions: *mesI*, immunity to mesentericin Y105; *mesY*, structural gene of mesentericin Y105; *mesC*, unknown; *mesDE*, secretion of mesentericins Y105 and B105; *mesF*, unknown; *mesH*, immunity to mesentericin B105; *mesB*, structural gene of mesentericin B105; and *mesG*, unknown.

In the confirmed plasmids, the nine genes (*mesIYCDEFHBG*) related to biosynthesis and immunity for mesentericins Y105 and B105 were searched for homology using the BLAST program (Figure 2). The sequences of the nine genes on plasmids pLM406A and pLM213M0A were 100% identical except for the functionally unknown *mesG*. The homology of *mesG* between them was 77%. This was because the middle region (60 bp) of *mesG* was missing on pLM213M0A, although the other region was 100% identical to that on pLM406A. The nine genes of both strains also had much higher similarity to those on plasmid pFR38 (accession no. AY286003) in *Leu. mesenteroides* FR52 (a producer of mesentericins 52A and 52B = mesentericins Y105 and B105) [26] compared with pHY30 (accession no. X81803 and AF143443) on strain Y105 (a producer of mesentericins Y105 and B105) [22,24,25]. In addition, the two transporter genes (*mesDE*) on pLM213M0B had lower homology to those on pFR38, pLM406A, and pLM213M0A (82 and 78%), and those on pHY30 (92 and 88%). These results suggested that differences in the production and antibacterial activity of bacteriocins between strains 406 and 213M0 might be due to shortened *mesG* and/or duplicated *mesDE* genes.

### 3.5. Plasmid Curing

**Figure 3:**
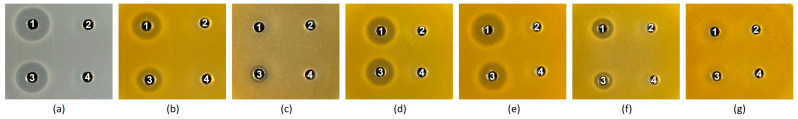
Antibacterial activity of *Leuconostoc mesenteroides* subsp. *mesenteroides* 406 (1) and 213M0 (3), and their derivative strains ((2) and (4) from 406 and 213M0, respectively), obtained by plasmid curing using novobiocin. Indicator strains: *Listeria monocytogenes* VTU 206 (**a**), *Weissella paramesenteroides* JCM 9890^T^ (**b**), *Leuconostoc lactis* JCM 6123^T^ (**c**), *Enterococcus faecalis* JCM 5803^T^ (**d**), *Leu. mesenteroides* subsp. *dextranicum* NBRC 100495^T^ (**e**), *Weissella cibaria* JCM 12495^T^ (**f**), and *Leu. mesenteroides* subsp. *cremoris* NBRC 107766^T^ (**g**).

**Figure 4:**
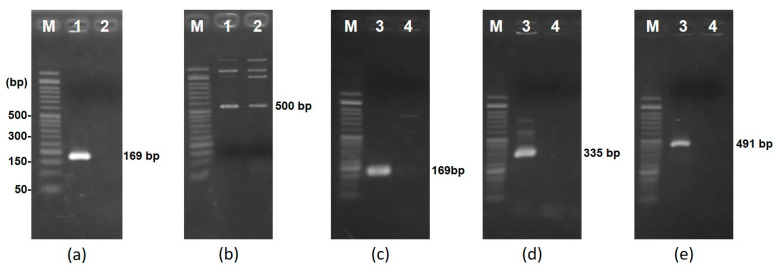
Profiles of plasmids pLM406A (**a**) and pLM406B (**b**) in *Leuconostoc mesenteroides* subsp. *mesenteroides* 406 (lane 1) and the derivative strain (lane 2) and pLM213M0A (**c**), pLM213M0B (**d**), and pLM213M0C (**e**) in *Leu. mesenteroides* subsp. *mesenteroides* 213M0 (lane 3) and the derivative strain (lane 4) using each plasmid-specific primer. Lane M: FastGene 50-bp DNA Ladder (Nippon Genetics Co.).

To investigate the source of the antibacterial activity of strains 406 and 213M0, plasmid curing was performed using novobiocin. Through dozens of subcultures, derivative strains without any antibacterial activity against the seven indicator strains were obtained (Figure 3). Then, their plasmid profiles were observed by agarose gel electrophoresis after PCR with the specific primers (Table 2) for each plasmid. As a result, it was confirmed that the derivative strain from 406 lost pLM406A but retained pLM406B (Figure 4a,b). On the other hand, the derivative strain from 213M0 lost all three plasmids, pLM213M0A, pLM213M0B, and pLM213C (Figure 4c–e). These results meant that the antibacterial activity of strains 406 and 213M0 was due to the bacteriocins encoded on the plasmids.

### 3.6. Purification of Bacteriocins

Bacteriocins produced by strains 406 and 213M0 were purified from their CFSs in three steps: ammonium sulfate precipitation, C18 solid-phase extraction, and C8 reverse-phase HPLC. The fractions obtained in each step were applied to peptide quantification and antibacterial activity assays against *Lis. monocytogenes* VTU 206, *W. paramesenteroides* JCM 9890^T^, and *Leu. lactis* JCM 6123^T^. The purification status at each step is shown in Supplementary Table S1.

**Figure 5:**
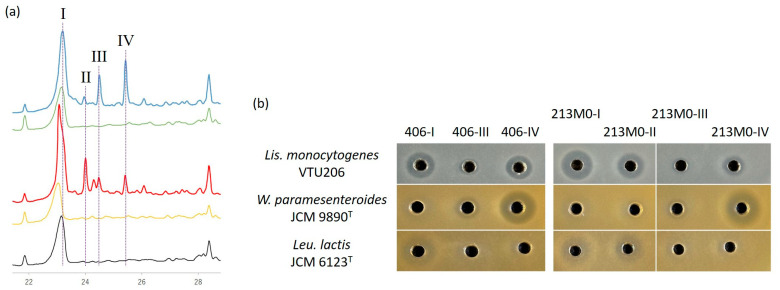
(**a**) HPLC chromatogram to purify bacteriocins produced by *Leuconostoc mesenteroides* subsp. *mesenteroides* 406 (blue) and 213M0 (red). The 406- and 213M0-derivative strains (green and yellow, respectively) without antibacterial activity, and MRS broth (black), were used as controls. The purified fractions around peaks-I, -III, and -IV in strain 406 and peaks-I, -II, -III, and -IV in strain 213M0 were collected. (**b**) Antibacterial activity of the three and four peak fractions (406-I, -III, and -IV and 213M0-I, -II, -III, and -IV) purified by HPLC against *Listeria monocytogenes* VTU 206, *Weissella paramesenteroides* JCM 9890^T^, and *Leuconostoc lactis* JCM 6123^T^.

In the final step using HPLC, three and four active peak fractions were collected from sample solutions of strains 406 and 213M0, respectively, while no active peaks were detected in samples of their derivative strains and MRS broth (control) (Figure 5a). For strain 406, the active fraction around peak-II was not collected because the peak was only a trace. Figure 5b and Supplementary Table S1 show the antibacterial activity of the collected fractions against three indicator strains. 

The peak-I fractions of both strains had higher activity than the other fractions against *Lis. monocytogenes* VTU 206 and *Leu. lactis* JCM 6123^T^ but no activity against *W. paramesenteroides* JCM 9890^T^. On the other hand, the peak-IV fractions had very high activity against *W. paramesenteroides* JCM 9890^T^ but no activity against *Leu. lactis* JCM 6123^T^. The peak-III fractions also showed a similar antibacterial spectrum to the peak-IV fractions, but the activity of the peak-III was much lower than that of the peak-IV. The activity of the peak-III and -IV fractions of strain 406 tended to be higher than that of the corresponding peak fractions of 213M0. This indicated that the production of the peak-III and -IV bacteriocins by strain 406 was high compared with that of 213M0.

Peak-II was detected only in the 213M0 sample (Figure 5a). This fraction had antibacterial activity against *Lis. monocytogenes* VTU 206 and *Leu. lactis* JCM 6123^T^ but no activity against *W. paramesenteroides* JCM 9890^T^ (Figure 5b). This spectrum was similar to that of the peak-I fraction. The peak-II fraction had less than one-fifth the activity and one-eighth the specific activity of the peak-I fraction against *Lis. monocytogenes* VTU 206 but more than half the activity and the specific activity of the peak-I fraction against *Leu. lactis* JCM 6123^T^ (Supplementary Table S1). This result suggested that the peak-II bacteriocin would be different from the peak-I as well as the peak-III and -IV bacteriocins and that only strain 213M0 would produce another bacteriocin that strain 406 did not produce.

### 3.7. Identification of Purified Bacteriocins

The purified bacteriocins in the three and four fractions from strains 406 and 213M0, respectively, were identified by N-terminal amino acid sequencing and MALDI-TOF MS analysis (Table 4 and Supplementary Figure S2). 

From the resulting N-terminal sequences and masses, the 406-I and 213M0-I bacteriocins were identified as mesentericin Y105 [21,24,30]. Although the measured masses ([M + H]^+^ = 3869) were two less than the expected mass ([M + H]^+^ = 3871) from the amino acid sequences, the value corresponded to that of mesentericin Y105 with a disulfide bridge as reported previously [31].

The 406-IV and 213M0-IV bacteriocins were a perfect match to mesentericin B105 [24,30] in both the resulting N-terminal sequences and masses. 

The N-terminal sequence of the 406-III bacteriocin was the same as that of mesentericin B105, but its molecular mass ([M + H]^+^ = 3463) corresponded to that of mesentericin B105 ([M + H]^+^ = 3447) plus 16. This indicated that the 406-III bacteriocin was the oxidized form of mesentericin B105 at one methionine residue [24,30], as has been well studied, especially with pediocin PA-1 produced by *Pediococcus acidilactici* strains [32,33,34]. The N-terminal amino acids of the 213M0-III bacteriocin could not be unambiguously sequenced because of contamination with (an)other peptide/protein(s). However, since its mass was measured to be the same as that of the 406-III bacteriocin, it was also considered to be oxidized mesentericin B105.

**Figure 6:**
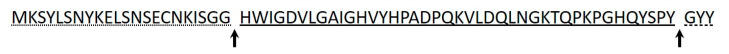
Sequence of mesentericin M (solid underlined) and the pre-peptide. Arrows refer to cleavage sites. Dotted and dashed underlined sequences indicate a signal peptide and a post-translationally released tripeptide, respectively.

The N-terminal sequence of the 213M0-II bacteriocin was HWIGDVLGAIGHV-. This was identical to a part of the translated sequence (66-aa, accession no. WP_061399677 and BFP62698) of a gene on pLM213M0B. In the translated sequence, upstream of the resulting sequence was a putative leader sequence (22-aa) with a double glycine motif at the C-terminus. The mass of the bacteriocin was predicted to be [M + H]^+^ = 4947.5 based on the translated sequence excluding the leader peptide, but was actually [M + H]^+^ = 4564 in the MALDI-TOF MS analysis. In order to investigate the cause of this discrepancy, MS/MS analysis followed by a Mascot search was next performed for the full-length sequence of the bacteriocin (Supplementary Figure S3 and Table S2). As a result, a 41-aa peptide sequence was obtained (Figure 6). Surprisingly, the resulting sequence had three C-terminal residues truncated from the translated sequence. To our knowledge, this is the first report of a C-terminal truncated bacteriocin. In addition, the full length of the sequence did not have high homology to any other bacteriocin sequences and was only partially close to some putative bacteriocins represented by infantaricin H (Supplementary Figure S4) [35]. These results indicate that mesentericin M is a novel type of bacteriocin with no relatives.

## 4. Discussion

*Leu. mesenteroides* subsp. *mesenteroides* 406 and 213M0 were both isolated from Mongolian traditional fermented milk, airag, and had similar antibacterial properties [17,18]. Genome information [19,20] had suggested that both strains produce the same bacteriocins, mesentericins Y105 and B105 [21,22,23,24,25,26,30,31]. In this study, it was actually shown, by molecular purification and subsequent analysis, that both strains produce them. Although some cases were given different names, mesentericins Y105 and B105 have been reported to be produced by many strains of *Leuconostoc* spp. such as *Leu. mesenteroides* SJRP55 isolated from Brazilian water buffalo mozzarella [36], *Leu. mesenteroides* subsp. *cremoris* W3 isolated from wine [37], and *Leu. mesenteroides* E131 isolated from Greek traditional fermented sausage [38]. This means that it is quite common for *Leuconostoc* spp. to possess the genes for production of mesentericins Y105 and B105 and that the genes are well conserved and have spread in various environments over a long evolutionary process.

However, the impetus to begin this study is the subtle differences in the antibacterial properties between strains 406 and 213M0. The antibacterial activity of strain 406 had been found to be higher than that of strain 213M0 against most target strains, and this was confirmed in this study (Table 1). On the other hand, a few exceptions were observed, with strain 213M0 being more active against *Lis. monocytogenes* JCM 7680 and *Leu. lactis* JCM 6123^T^ (Table 1). This reversal was attributed to the presence of mesentericin M, produced only by strain 213M0, that was revealed for the first time in this study. Mesentericin M is a novel type of bacteriocin with no highly homologous bacteriocins, although partially homologous putative bacteriocins such as infantaricin H produced by *Streptococcus infantarius* LP90 have been reported (Supplementary Figure S4) [35]. Furthermore, mesentericin M is, to our knowledge, the first bacteriocin in which the C-terminal three amino acid residues (GYY) are post-translationally cleaved. One of the identified bacteriocins that underwent a somewhat similar post-translational modification at the C-terminus was microcin E492, produced by *Klebsiella pneumoniae* RYC492, but it was not cleaved [39]. After translation, the pre-peptide of microcin E492 was modified at the C-terminal serine by the addition of a C-glycosylated trimer of N-(2,3-dihydroxybenzoyl)-L-serine to form the mature bacteriocin with stronger antibacterial activity. This process requires a glycosyltransferase (*mceC*) and an enterobactin esterase (*mceD*) encoded on the microcin E492 gene cluster (*mceABCDEHGHIJ*).

**Figure 7:**
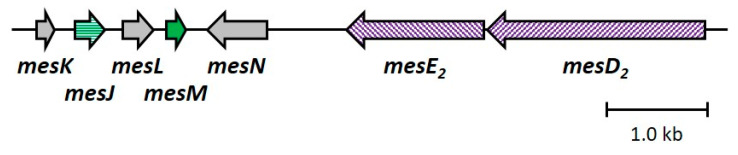
A deduced mesentericin M-related gene cluster on pLM213M0B. Gene functions: *mesK*, unknown; *mesJ*, immunity to mesentericin M; *mesL*, unknown; *mesM*, structural gene of mesentericin M; *mesN*, unknown; *mesE_2_D_2_*, secretion of mesentericin M.

Based on the sequence information of mesentericin M revealed in this study, we here infer the genes responsible for its biosynthesis and immunity around its structural gene (*mesM*) on pLM213M0B (Supplementary Figure S1d and Figure 7). There are three genes (named *mesKJL*) and one gene (*mesN*) upstream and downstream near *mesM*, respectively. Among them, *mesJ* was annotated as a putative bacteriocin immunity gene, and its upstream neighbor is *mesK*. Although the function of *mesK* is unknown, a sequence with 98% homology to *mesK* was found in the vicinity of the ABC transporter genes (*lcaCD*) of leucocin A produced by *Leuconostoc gelidum* UAL 187 [7]. There is a putative promoter just upstream of *mesK*, and there is a replication initiation gene that is not directly related to bacteriocin production and immunity in further approximately 800 bp upstream. Therefore, *mesK* is thought to be the first gene of the mesentericin M-related gene cluster. Downstream of *mesM* is *mesN*, whose function is unknown, and approximately 770 bp further downstream is *mesD_2_E_2_*, described as pLM213M0B-*mesDE* in Figure 2. These two genes had been thought to be responsible for the secretion of mesentericins Y105 and B105, as mentioned above. However, since there are no other mesentericin M secretion genes in the vicinity, it was speculated that they would play this role. On these grounds, these seven genes were deduced to be the mesentericin M-related genes.

As noted above, mesentericin M requires not only cleavage of the N-terminal leader peptide but also cleavage of the C-terminal tripeptide for its maturation. For cleavage at the C-terminus, some kind of protease such as a carboxypeptidase would be needed, as well as a signal peptidase at the N-terminus. The three genes (*mesK*, *mesL,* and *mesN*) of unknown function on pLM213M0B may possibly play a role in this protease activity. However, this seems unlikely, since the translated sequences of *mesK*, *mesL,* and *mesN* do not have the well-known conserved regions of proteases. We rather focus on the homology between the cleaved tripeptide (GYY) and the first three residues (KYY) on the N-terminal side (just after the double glycine motif in the leader sequence) of mesentericin Y105. Namely, we think that the leader peptidase of mesentericin Y105 may misrecognize and cleave the tripeptide. Further experiments are needed to elucidate the cleavage mechanism of the C-terminal tripeptide and its effects.

As for the 406-III bacteriocin, the N-terminal sequence was identical to that of mesentericin B105 (Table 4). However, its mass, as well as that of the 123M0-III bacteriocin, was 16 higher than that of mesentericin B105, identified in the peak-IV fraction (Supplementary Figure S2). This was consistent with the value obtained by oxidation at the ninth amino acid residue, methionine, to sulfoxide of mesentericin B105. Similar methionine oxidation has been well studied with pediocin PA-1 [32,33,34]. It has been reported that the methionine residue at position 31 of pediocin PA-1 was gradually oxidized to sulfoxide during storage and that the antibacterial activity was greatly reduced by the oxidation. The specific activities of the peak-III bacteriocins were also significantly lower than those of the corresponding peak-IV bacteriocins identified as mesentericin B105 (Figure 5b and Supplementary Table S1). These results indicated that the peak-III bacteriocins were oxidized mesentericin B105, whereas any active peaks of the oxidized forms of mesentericins Y105 and M were not detected (Figure 5a), which must be due to the absence of a methionine residue in them. Incidentally, almost the same results were also observed for mesentericin B105 and 52B [24,30].

As shown in Figure 5 and Supplementary Table S1, mesentericins Y105 and M had no antibacterial activity, but mesentericin B105 and the oxidized form had activity against *W. paramesenteroides* JCM 9890^T^. The activity of strain 406 against strain JCM 9890^T^ was significantly higher than that of strain 213M0, meaning that strain 406 produced more mesentericin B105 than 213M0. In fact, the amount of mesentericin B105 purified from the 406 culture was approximately twice that from the 213M0 culture (Supplementary Table S1). On the other hand, there was not much difference in the amount of mesentericin Y105 produced between strains 406 and 213M0 (Supplementary Table S1). The difference in the production of mesentericin B105, despite roughly equal production of mesentericin Y105, is one of the factors contributing to the generally higher antibacterial activity of strain 406 compared with strain 213M0. The comparison of production levels also suggests that, unlike mesentericin Y105, the production of mesentericin B105 by strain 213M0 would not reach its maximum. The nucleotide sequence comparison (Figure 2) in this study showed that the genetic differences between strains 406 and 213M0 to produce mesentericins Y105 and B105 were the shortening of *mesG* and the duplication of the transporter genes (*mesDE* and *mesD_2_E_2_*). Although *mesG* is rumored to encode an additional putative accessory protein [40], it has no significant similarity to other already-known functional protein-coding genes [25]. In Morisset and Frère (2002), the mutant strains expressing the eight *mes* genes (*mesIYCDEFHB*), except *mesG,* produced mesentericin B105 at least as much as the wild strain Y105 [41]. In addition, no effect of *mesG* on the primary structure of mesentericin B105 was detected by mass analysis in this study (Table 4 and Supplementary Figure S2). Therefore, there may be no special function for *mesG*. In other words, the shortening of *mesG* did not seem to affect the low production or the primary structure of mesentericin B105 in strain 213M0. Another possible influencing factor, the duplication of similar secretory genes (*mesDE* and *mesD_2_E_2_*), might lead to decreased production of mesentericin B105. The leader sequence of mesentericin M is more like that of mesentericin B105 (50.0%) than that of mesentericin Y105 (33.3%). Such leader sequence similarity might lead to transporter competition, resulting in a decrease in mesentericin B105 secretion instead of an increase in mesentericin M secretion. The effects of the *mesG* incompleteness and transporter duplication on mesentericin production are only predictions, and thus require further study.

In strain 406, plasmid pLM406B, harboring several genes involved in citrate metabolism (Supplementary Figure S1b), was found apart from mesentericin-related pLM406A. It is well known that citrate is a growth promoter that produces ATP for LAB strains with citrate-metabolizing ability, allowing them to grow even in environments lacking fermentable carbohydrates [42]. In this study, strain 406 grew better than strain 213M0 (Figure 1), which might be due to the pLM406B-originated metabolism of the citrate contained in MRS broth. However, the growth advantage of strain 406 did not appear to affect the difference in its bacteriocin production from strain 213M0. This can be seen from the result that there was not much difference in the production and activity of the purified mesentericin Y105 (Supplementary Table S1).

In conclusion, it was revealed that *Leu. mesenteroides* subsp. *mesenteroides* 406 and 213M0 both produced plasmid-encoded bacteriocins, mesentericins Y105 and B105 (including oxidized B105), and that only strain 213M0 produced a novel C-terminal tripeptide-truncated bacteriocin, named as mesentericin M, without high homology to any other known bacteriocins. The differences in the antibacterial properties between strains 406 and 213M0 should have resulted not only from the presence of mesentericin M, but also from the different amount of mesentericin B105, not mesentericin Y105. The findings in this comparative study would greatly contribute to the future use of these two strains and their bacteriocins as food biopreservatives and fermentation controllers, especially to inhibit *Listeria* contamination.

## Figures and Tables

**Table 1:** Bacterial strains and culture conditions used for the antibacterial activity assay and the resulting antibacterial spectra of *Leuconostoc mesenteroides* subsp. *mesenteroides* 406 and 213M0.

Tested Bacterium		Culture Condition ^2^	Antibacterial Activity (AU/mL) ^3^	
Species	Strain ^1^		Strain 406	Strain 213M0
*Listeria monocytogenes*	VTU 206	37 °C, TYLG	4969 ± 99.1	2695 ± 63.1
*Listeria monocytogenes*	JCM 7671	37 °C, TYLG	3093 ± 111.6	2560 ± 105.2
*Listeria monocytogenes*	JCM 7672	37 °C, TYLG	3328 ± 85.9	3072 ± 97.1
*Listeria monocytogenes*	JCM 7673	37 °C, TYLG	3584 ± 70.1	2944 ± 105.2
*Listeria monocytogenes*	JCM7674	37 °C, TYLG	5546 ± 188.4	3584 ± 105.2
*Listeria monocytogenes*	JCM 7675	37 °C, TYLG	2560 ± 0.0	2560 ± 0.0
*Listeria monocytogenes*	JCM 7679	37 °C, TYLG	5120 ± 0.0	5120 ± 0.0
*Listeria monocytogenes*	JCM 7680	37 °C, TYLG	1706 ± 37.0	2560 ± 0.0
*Listeria ivanovii* subsp. *ivanovii*	JCM 7681^T^	37 °C, TYLG	15,360 ± 280.4	9386 ± 104.5
*Enterococcus faecalis*	JCM 5803^T^	37 °C, MRS	724 ± 13.6	600 ± 6.8
*Enterococcus faecium*	JCM 5804^T^	37 °C, MRS	-	-
*Lactococcus cremoris* subsp. *cremoris*	NBRC 100676^T^	30 °C, MRS	-	-
*Lactococcus lactis* subsp. *lactis*	NBRC 100933^T^	30 °C, MRS	-	-
*Lactococcus lactis* subsp. *lactis*	IFO 12007	30 °C, MRS	-	-
*Lactococcus lactis* subsp. *lactis*	NIAI 527	30 °C, MRS	-	-
*Lactococcus lactis* subsp. *lactis*	NIAI N-7	30 °C, MRS	-	-
*Lactococcus lactis* subsp. *lactis*	JCM 7638	30 °C, MRS	-	-
*Lactococcus lactis* subsp. *hordniae*	NBRC 100931^T^	30 °C, MRS	-	-
*Leuconostoc mesenteroides* subsp. *cremoris*	NBRC 107766^T^	30 °C, MRS	72 ± 2.5	32 ± 1.3
*Leuconostoc mesenteroides* subsp. *dextranicum*	NBRC 100495^T^	30 °C, MRS	471 ± 11.2	235 ± 5.5
*Leuconostoc mesenteroides* subsp. *mesenteroides*	NBRC 100496^T^	30 °C, MRS	-	-
*Leuconostoc lactis*	JCM 6123^T^	30 °C, MRS	20 ± 0.0	40 ± 1.4
*Pediococcus acidilactici*	JCM 8797^T^	30 °C, MRS	-	-
*Pediococcus parvulus*	JCM 5889^T^	30 °C, MRS	-	-
*Pediococcus pentosaceus*	JCM 5885	37 °C, MRS	-	-
*Pediococcus pentosaceus*	JCM 5890^T^	37 °C, MRS	-	-
*Streptococcus thermophilus*	JCM 17834^T^	37 °C, MRS	-	-
*Streptococcus thermophilus*	JCM 20026	37 °C, MRS	-	-
*Weissella cibaria*	JCM 12495^T^	30 °C, MRS	297 ± 8.6	114 ± 6.1
*Weissella confusa*	JCM 1093^T^	30 °C, MRS	-	-
*Weissella paramesenteroides*	JCM 9890^T^	30 °C, MRS	1768 ± 30.3	522 ± 8.4
*Weissella viridescens*	JCM 1174^T^	37 °C, MRS	-	-

^1^ VTU, Department of Veterinary Public Health, University of Tokyo; JCM, Japan Collection of Microorganisms; NBRC, NITE Biological Resource Center, Japan; IFO, Institute for Fermentation, Osaka; and NIAI, National Institute of Animal Industry, Japan. ^2^ TYLG, Tryptone, yeast extract, lactose, and glucose broth [27]; and MRS, de Man, Rogosa, and Sharpe broth. ^3^ AU, arbitrary units (mean ± S.D.); and -, no inhibition.

**Table 2:** Primers used in this study.

Primer Name	Sequence (5′–3′)	Target
406-33-F1	CCCAATACACCTTTACCACCAC	Contig-33 (pLM406A) in strain 406
406-33-R1	CTTGGATTGTGGGAACAAGA	
406-40-F1	AGAAACTGCCCGTGATGGAAAC	Contig-40 (pLM406B) in strain 406
406-40-R1	GCTGGTGTTGGATTGTCTTTGCT	
213-26-F4	AGCGGTTGCTATAACGGCTA	Contig-26 (pLM213M0A) in strain 213M0
213-26-R4	GCTTCAAATGACGACTGCAA	
213-26-F5	CGAGCTTTAAAGGGTGCTGAAAAAT	Contig-26 (pLM213M0A) in strain 213M0
213-26-R5	CGCTACTGAATTTCTTGTCAAGGTTGT	
213-30-F1	TTAGTCCGTGAGCGGTTTATGAGAG	Contig-30 (pLM213M0B) in strain 213M0
213-30-R1	AATCAAGAAAGGAGCTGTGATGACG	
213-48-F2	TTGCGCTAATCGGTCAATGG	Contig-48 (pLM213M0C) in strain 213M0
213-48-R2	GTGACCGACCGTAGGGAGACTTTAT	
mesY-F	ACCAAAATCCATTTCCACCA	Structural gene (*mesY*) of mesentericin Y105
mesY-R	TCTGTGGAAGCATATCAGCAA	

**Table 3:** Overview of plasmids in *Leuconostoc mesenteroides* subsp. *mesenteroides* 406 and 213M0.

Plasmid	Length (bp)	GC (%)	ORFs	Accession No.
pLM406A	32,775	34.2	42	LC832860
pLM406B	23,705	39.4	24	LC832861
pLM213M0A	41,975	33.5	51	LC832857
pLM213M0B	11,669	33.8	14	LC832858
pLM213M0C	6449	30.2	8	LC832859

**Table 4:** N-terminal sequences and molecular masses of active peptides purified stepwise from the cultures of *Leuconostoc mesenteroides* subsp. *mesenteroides* 406 and 213M0.

Producer	Fraction	N-Terminal Sequence	MS [M + H]^+^	Identification
*Leuconostoc* *mesenteroides* 406	406-I	KYYGNGVH(X)TKSG-	3869	Mesentericin Y105
	406-III	KGVLGWLSMASSA-	3463	Oxidized mesentericin B105
	406-IV	KGVLGWL-	3447	Mesentericin B105
*Leuconostoc* *mesenteroides* 213M0	213M0-I	KYYGNGVH(X)TKSG-	3869	Mesentericin Y105
	213M0-II	HWIGDVLGAIGHV-	4564	Mesentericin M ^1^
	213M0-III	-	3463	Oxidized mesentericin B105
	213M0-IV	KGVLGWL-	3447	Mesentericin B105

^1^ Novel bacteriocin identified in this study.

## Data Availability

Genetic data from this study have been deposited to DDBJ under accession numbers LC832857–LC832861. The other data in this study are available from the corresponding author upon reasonable request.

## Supplementary Materials

The following supporting information can be downloaded at: https://www.mdpi.com/article/10.3390/microorganisms12091781/s1, Figure S1: Maps of plasmids pLM406A (a) and pLM406B (b) in *Leuconostoc mesenteroides* subsp. *mesenteroides* 406 and pLM213M0A (c), pLM213M0B (d), and pLM213M0C (e) in *Leu. mesenteroides* subsp. *mesenteroides* 213M0; Table S1: Purification status of bacteriocins produced by *Leuconostoc mesenteroides* subsp. *mesenteroides* 406 and 213M0; Figure S2: MALDI-TOF-MS spectra of purified bacteriocins produced by *Leuconostoc mesenteroides* subsp. *mesenteroides* 406 and 213M0; Figure S3: MS/MS spectra of mesentericin M; Table S2: Monoisotopic mass (*m/z*) of fragment *a*-, *b*-, and *y*-ions in MS/MS spectra of mesentericin M; Figure S4: Sequence alignment of mesentericin M and infantaricin H.
