# Supplementary material for: A Novel C-Terminal Truncated Bacteriocin Found by Comparison between Leuconostoc mesenteroides 406 and 213M0 Isolated from Mongolian Traditional Fermented Milk, Airag
Source: Microorganisms. 2024 Aug 28;12(9):1781. doi: 10.3390/microorganisms12091781 (PMC11433673; doi:10.3390/microorganisms12091781)
Supplement: Supplementary file 1 [file microorganisms-12-01781-s001.zip › microorganisms-3116584-supplementary.pdf]

(a)

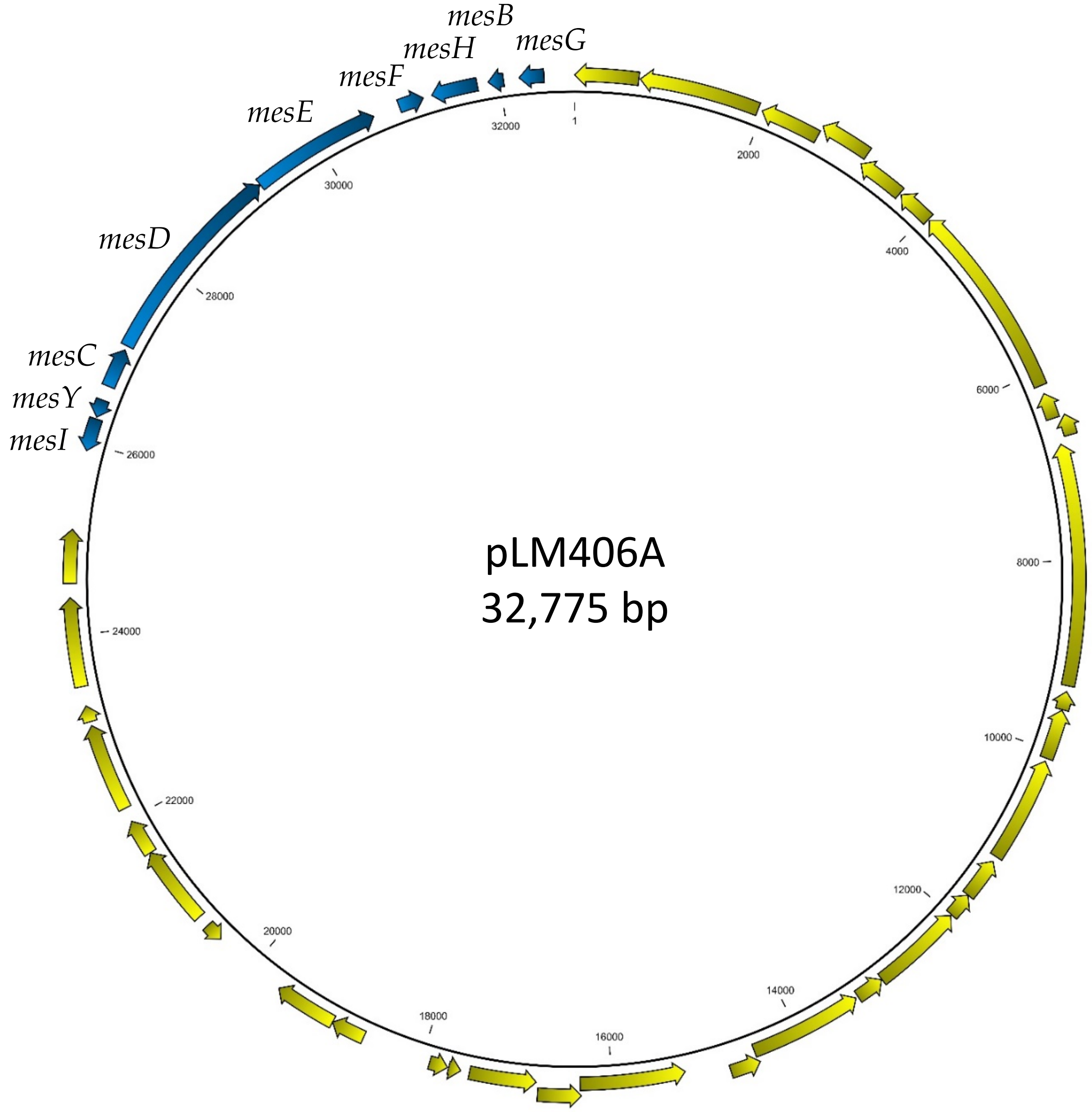

Supplementary Figure S1. Maps of plasmids pLM406A (a) and pLM406B (b) in *Leuconostoc mesenteroides* subsp. *mesenteroides* 406, and pLM213M0A (c), pLM213M0B (d) and pLM213M0C (e) in *Leu. mesenteroides* subsp. *mesenteroides* 213M0. Blue arrows indicate mesentericin Y105-B105-related genes. Green arrows indicate mesentericin M-related genes newly estimated in this study. Yellow allows are others.

(b)

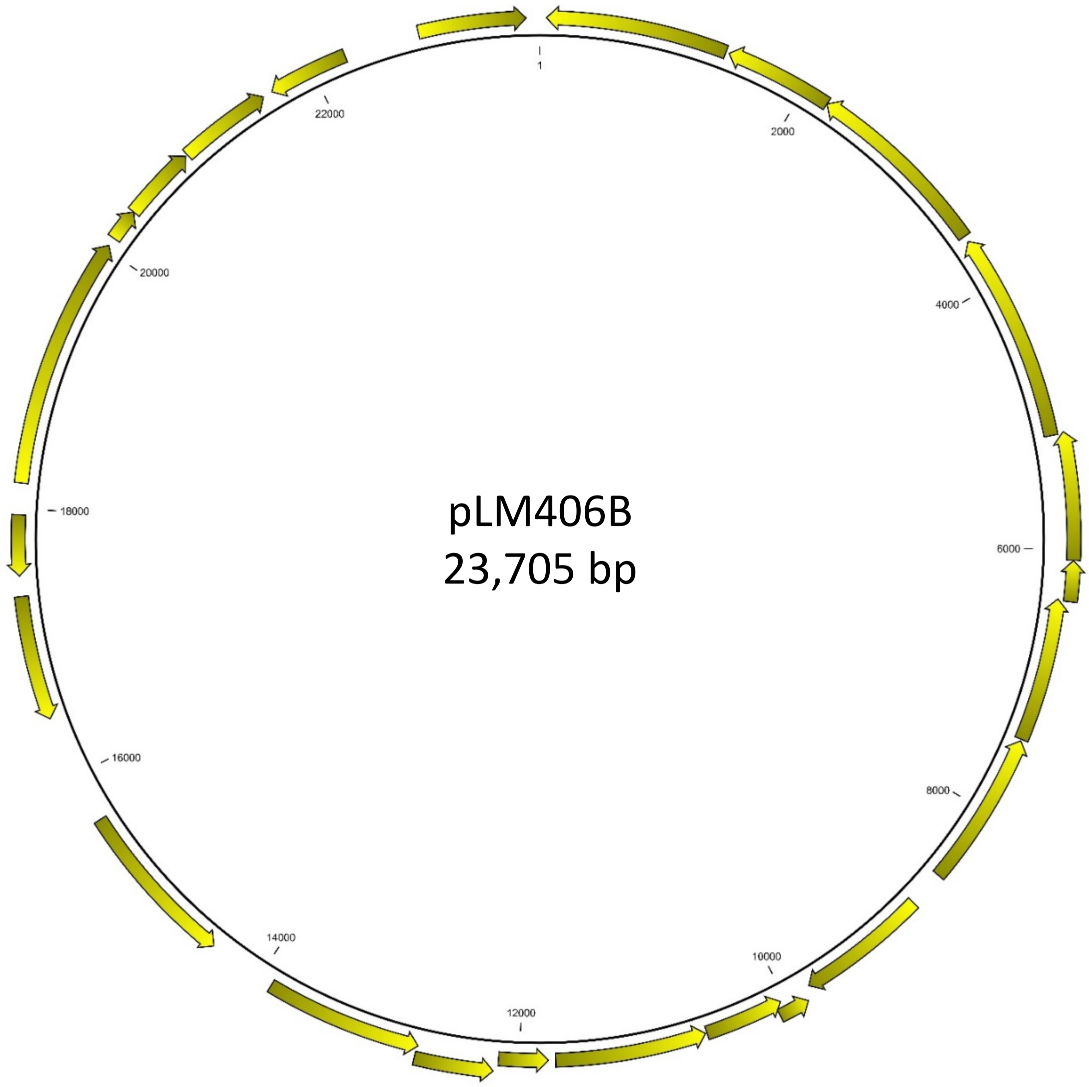

Supplementary Figure S1. Maps of plasmids pLM406A (a) and pLM406B (b) in *Leuconostoc mesenteroides* subsp. *mesenteroides* 406, and pLM213M0A (c), pLM213M0B (d) and pLM213M0C (e) in *Leu. mesenteroides* subsp. *mesenteroides* 213M0. Blue arrows indicate mesentericin Y105-B105-related genes. Green arrows indicate mesentericin M-related genes newly estimated in this study. Yellow allows are others.

(c)

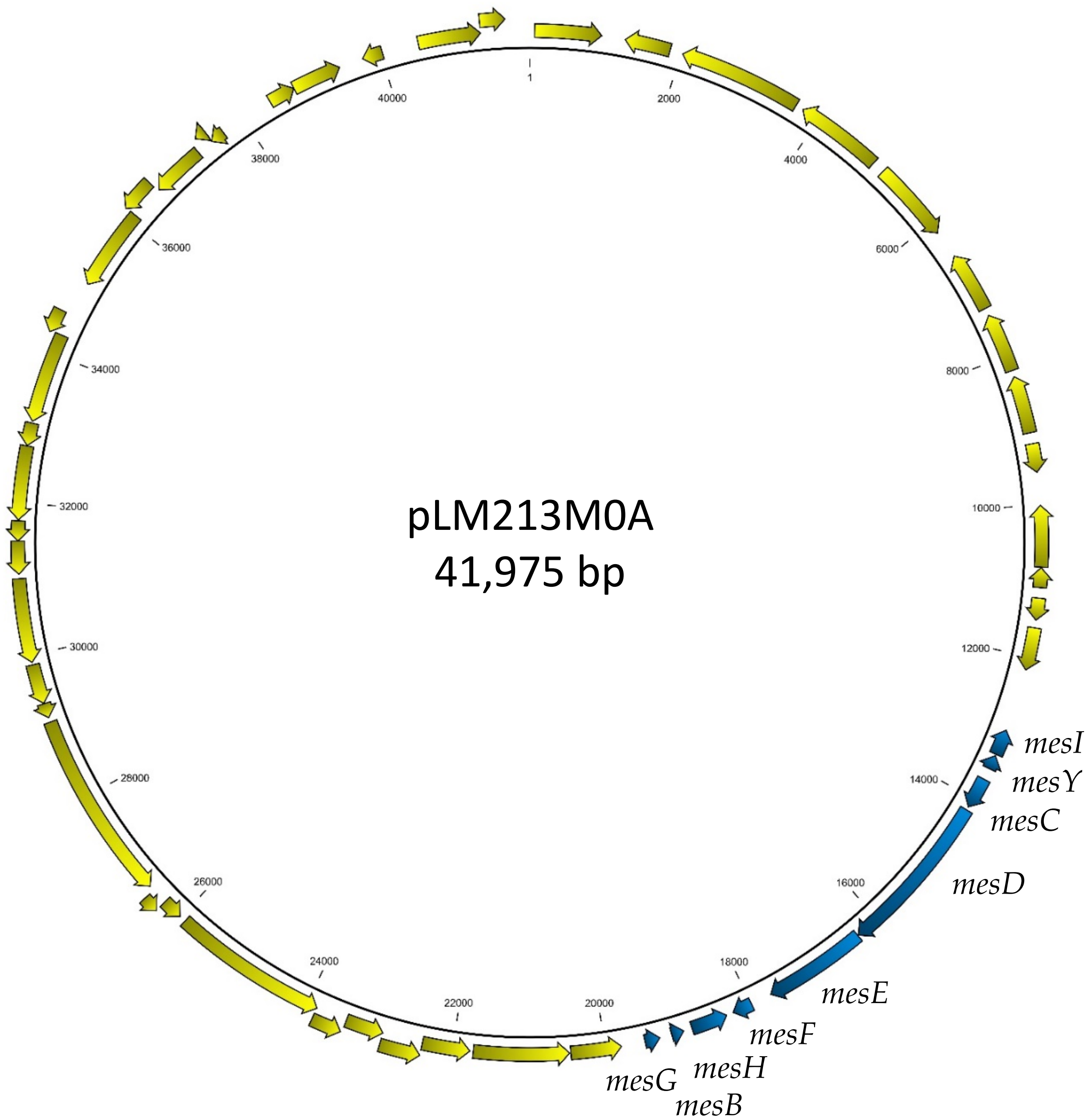

Supplementary Figure S1. Maps of plasmids pLM406A (a) and pLM406B (b) in *Leuconostoc mesenteroides* subsp. *mesenteroides* 406, and pLM213M0A (c), pLM213M0B (d) and pLM213M0C (e) in *Leu. mesenteroides* subsp. *mesenteroides* 213M0. Blue arrows indicate mesentericin Y105-B105-related genes. Green arrows indicate mesentericin M-related genes newly estimated in this study. Yellow allows are others.

(d)

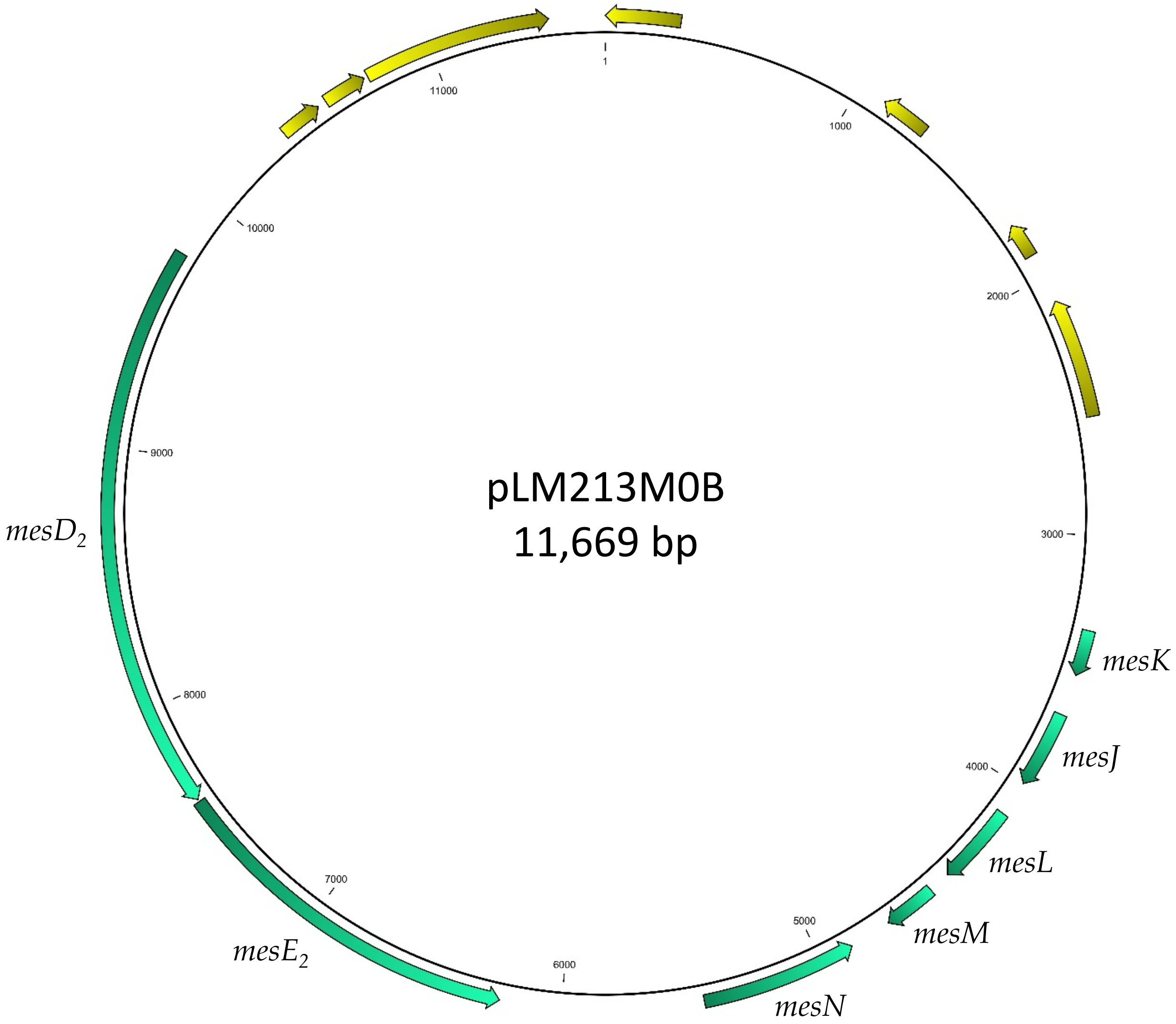

Supplementary Figure S1. Maps of plasmids pLM406A (a) and pLM406B (b) in *Leuconostoc mesenteroides* subsp. *mesenteroides* 406, and pLM213M0A (c), pLM213M0B (d) and pLM213M0C (e) in *Leu. mesenteroides* subsp. *mesenteroides* 213M0. Blue arrows indicate mesentericin Y105-B105-related genes. Green arrows indicate mesentericin M-related genes newly estimated in this study. Yellow allows are others.

(e)

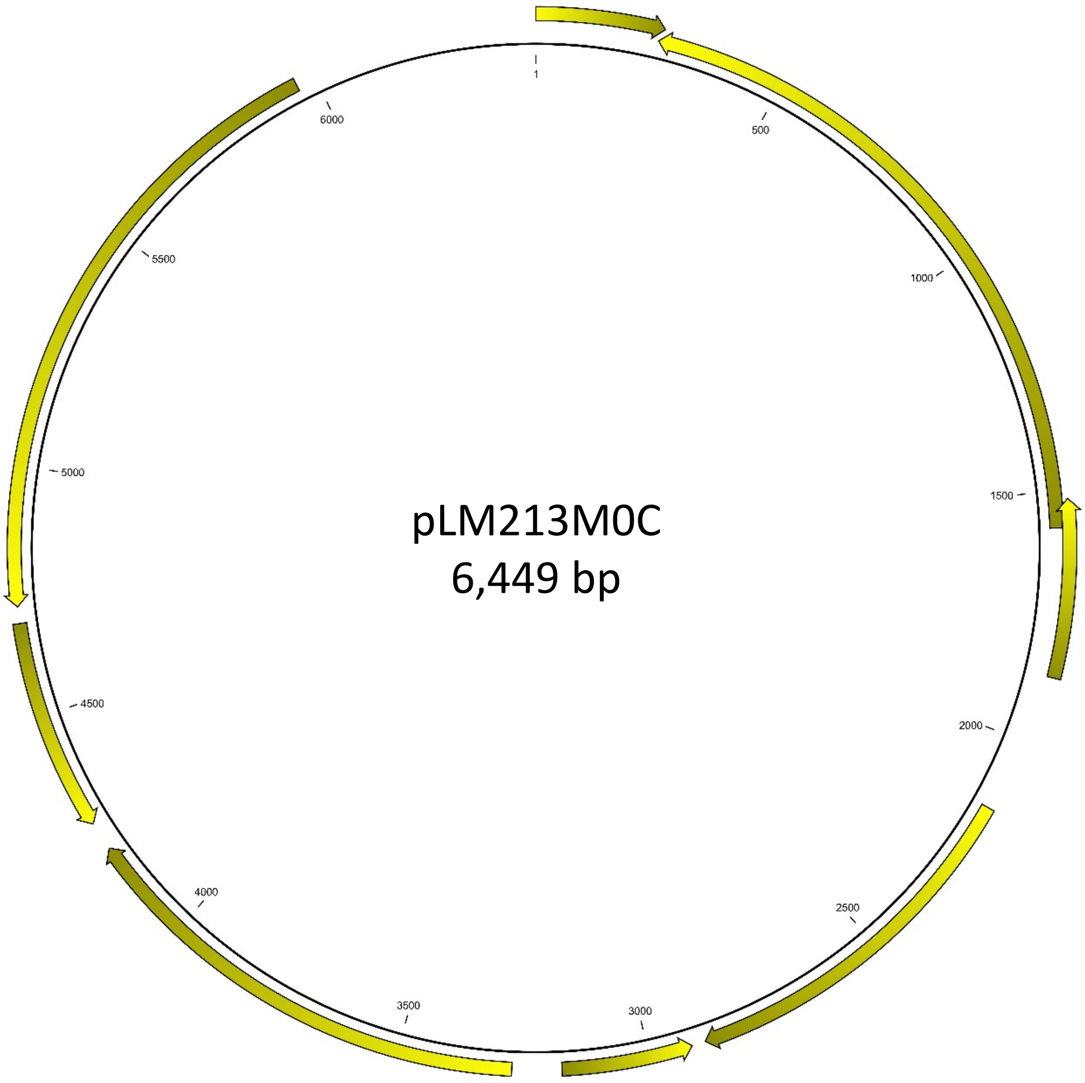

Supplementary Figure S1. Maps of plasmids pLM406A (a) and pLM406B (b) in *Leuconostoc mesenteroides* subsp. *mesenteroides* 406, and pLM213M0A (c), pLM213M0B (d) and pLM213M0C (e) in *Leu. mesenteroides* subsp. *mesenteroides* 213M0. Blue arrows indicate mesentericin Y105-B105-related genes. Green arrows indicate mesentericin M-related genes newly estimated in this study. Yellow allows are others.

Supplementary Table S1. Purification status of bacteriocins produced by *Leuconostoc mesenteroides* subsp. *mesenteroides* 406 and 213M0.

Sample	Purification step	Volume (mL)	Total protein (mg)	Activity (AU/mL)	Total activity (AU)	Activity recovered (%)	Specific activity (AU/mg)	Purification (fold)
**Activity against *Listeria monocytogenes* VTU 206**								
406	supernatant	100	1,116.58	2,880.00	288,000.00	100.00	257.93	1.00
	precipitation in $(NH_4)_2SO_4$	10	19.72	17,066.67	170,666.67	59.26	8,653.08	33.55
	sep-pak C18 (40% ACN)	5	0.1942	13,084.44	65,422.22	22.72	336,856.74	1,306.00
	C8 RP-HPLC (peak-I)	1	0.0026	1,813.33	1,813.33	0.63	685,479.12	2,657.61
	C8 RP-HPLC (peak-III)	1	0.0028	113.33	113.33	0.04	40,910.16	158.61
	C8 RP-HPLC (peak-IV)	1	0.0045	388.57	388.57	0.13	86,988.84	337.26
213M0	supernatant	100	1,224.10	2,400.00	240,000.00	100.00	196.06	1.00
	precipitation in $(NH_4)_2SO_4$	10	21.28	16,497.78	164,977.78	68.74	7,752.58	39.54
	sep-pak C18 (40% ACN)	5	0.1955	11,946.67	59,733.33	24.89	305,536.11	1,558.36
	C8 RP-HPLC (peak-I)	1	0.0033	1,554.29	1,554.29	0.65	474,785.85	2,421.61
	C8 RP-HPLC (peak-II)	1	0.0050	270.00	270.00	0.11	54,455.45	277.75
	C8 RP-HPLC (peak-III)	1	0.0021	120.00	120.00	0.05	57,191.08	291.70
	C8 RP-HPLC (peak-IV)	1	0.0021	220.00	220.00	0.09	103,704.20	528.94
**Activity against *Weissella paramesenteroides* JCM 9890^T**								
Sample	Purification steps	Volume (mL)	Total protein (mg)	Activity （AU/mL)	Total activity (AU）	Activity recovered (%)	Specific activity (AU/mg)	Purification (fold)
406	supernatant	100	1,116.58	1,780.00	178,000.00	100.00	159.42	1.00
	precipitation in $(NH_4)_2SO_4$	10	19.72	13,653.33	136,533.33	76.70	6,922.47	43.42
	sep-pak C18 (40% ACN)	5	0.1942	2,880.00	14,400.00	8.09	74,145.10	465.11
	C8 RP-HPLC (peak-I)	1	0.0026	-	-	-	-	-
	C8 RP-HPLC (peak-III)	1	0.0028	262.22	262.22	0.15	94,654.87	593.76
	C8 RP-HPLC (peak-IV)	1	0.0045	710.00	710.00	0.40	158,946.53	997.06
213M0	supernatant	100	1,224.10	530.00	53,000.00	100.00	43.30	1.00
	precipitation in $(NH_4)_2SO_4$	10	21.28	4,551.11	45,511.11	85.87	2,138.64	49.39
	sep-pak C18 (40% ACN)	5	0.1955	920.00	4,600.00	8.68	23,529.01	543.43
	C8 RP-HPLC (peak-I)	1	0.0033	-	-	-	-	-
	C8 RP-HPLC (peak-II)	1	0.0050	-	-	-	-	-
	C8 RP-HPLC (peak-III)	1	0.0021	115.56	115.56	0.22	55,072.89	1,271.98
	C8 RP-HPLC (peak-IV)	1	0.0021	188.89	188.89	0.36	89,038.96	2,056.47
**Activity against *Leuconostoc lactis* JCM 6123^T**								
Sample	Purification steps	Volume (mL)	Total protein (mg)	Activity （AU/mL)	Total activity (AU）	Activity recovered (%)	Specific activity (AU/mg)	Purification (fold)
406	supernatant	100	1,116.58	12.50	1,250.00	100.00	1.12	1.00
	precipitation in $(NH_4)_2SO_4$	10	19.72	120.00	1,200.00	96.00	60.84	54.35
	sep-pak C18 (40% ACN)	5	0.1942	186.67	933.33	74.67	4,805.70	4,292.76
	C8 RP-HPLC (peak-I)	1	0.0026	33.33	33.33	2.67	12,600.72	11,255.77
	C8 RP-HPLC (peak-III)	1	0.0028	-	-	-	-	-
	C8 RP-HPLC (peak-IV)	1	0.0045	-	-	-	-	-
213M0	supernatant	100	1,224.10	68.57	6,857.14	100.00	5.60	1.00
	precipitation in $(NH_4)_2SO_4$	10	21.28	400.00	4,000.00	58.33	187.97	33.55
	sep-pak C18 (40% ACN)	5	0.1955	266.67	1,333.33	19.44	6,820.00	1,217.47
	C8 RP-HPLC (peak-I)	1	0.0033	53.33	53.33	0.78	16,291.67	2,908.31
	C8 RP-HPLC (peak-II)	1	0.0050	45.00	45.00	0.66	9,075.91	1,620.19
	C8 RP-HPLC (peak-III)	1	0.0021	-	-	-	-	-
	C8 RP-HPLC (peak-IV)	1	0.0021	-	-	-	-	-

-, No activity.

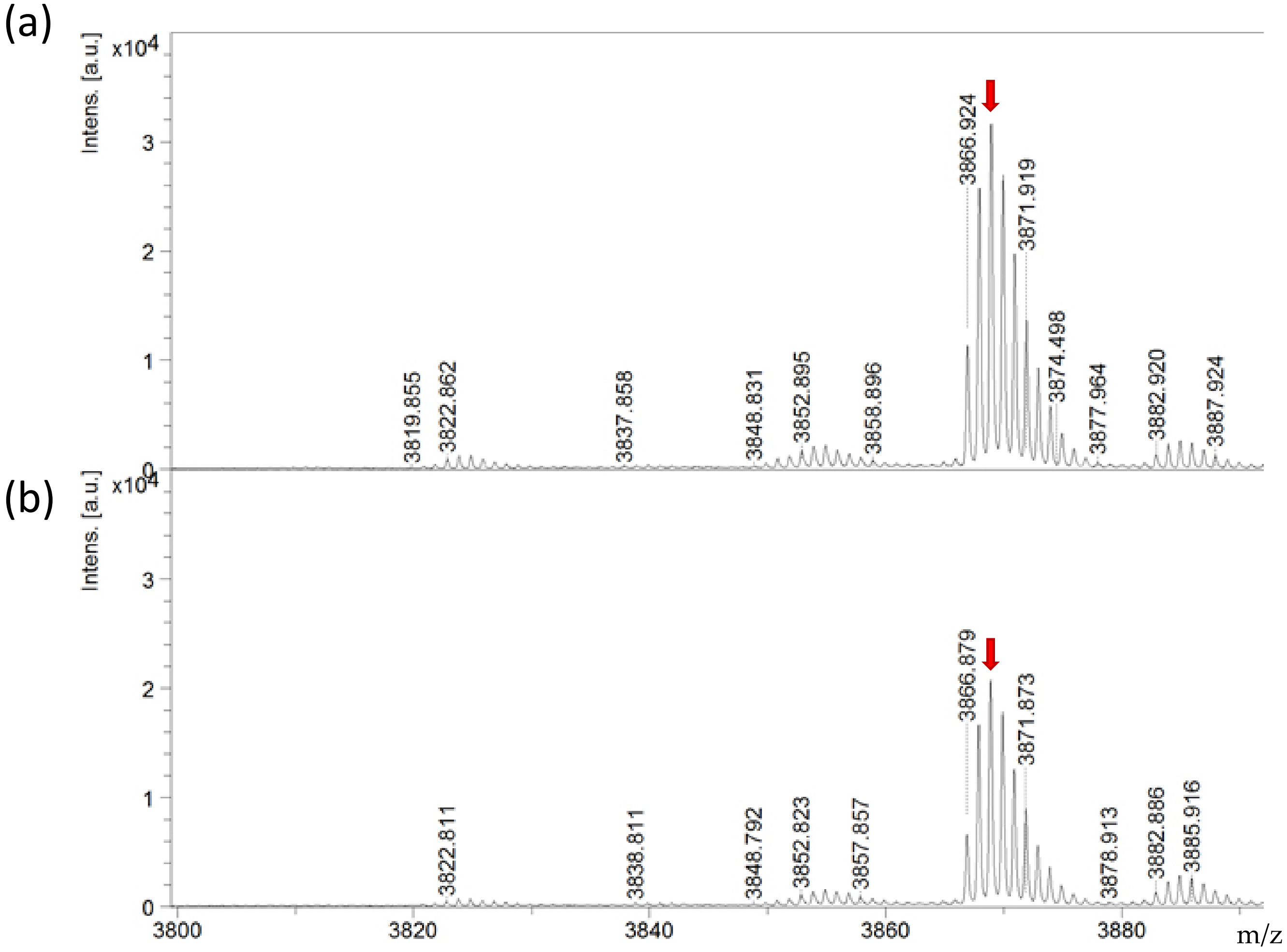

Supplementary Figure S2. MALDI-TOF-MS spectra of purified bacteriocins produced by *Leuconostoc mesenteroides* 406 and 213M0. Samples: peak-I of 406 (a), peak-I of 213M0 (b), peak-III of 406 (c1, c2), peak-III of 213M0 (d), peak-IV of 406 (e), peak-IV of 213M0 (f), peak-II of 213M0 (g), and blank (h). Red arrows indicate the highest peak in each analysis.

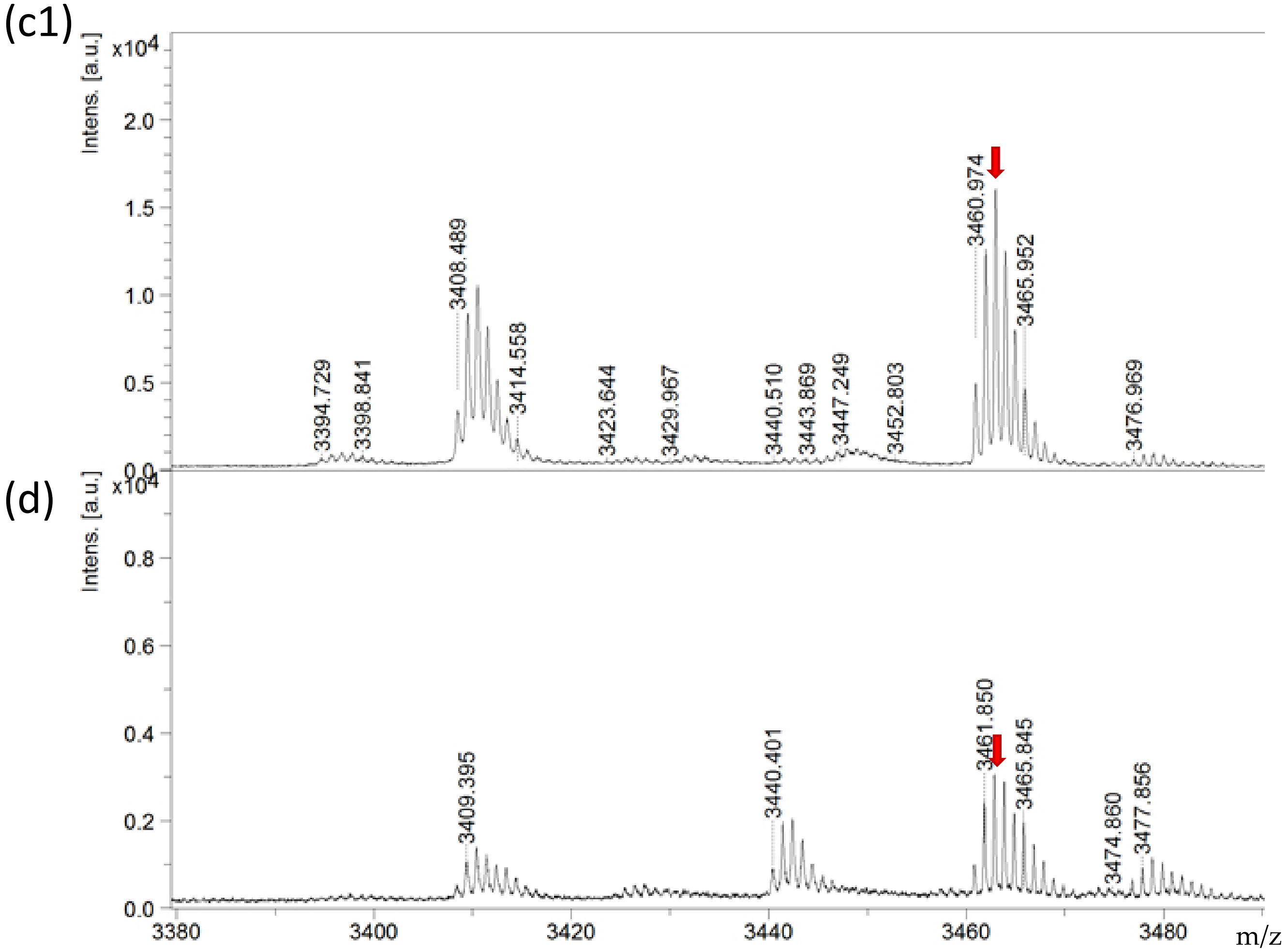

Supplementary Figure S2. MALDI-TOF-MS spectra of purified bacteriocins produced by *Leuconostoc mesenteroides* 406 and 213M0. Samples: peak-I of 406 (a), peak-I of 213M0 (b), peak-III of 406 (c1, c2), peak-III of 213M0 (d), peak-IV of 406 (e), peak-IV of 213M0 (f), peak-II of 213M0 (g), and blank (h). Red arrows indicate the highest peak in each analysis.

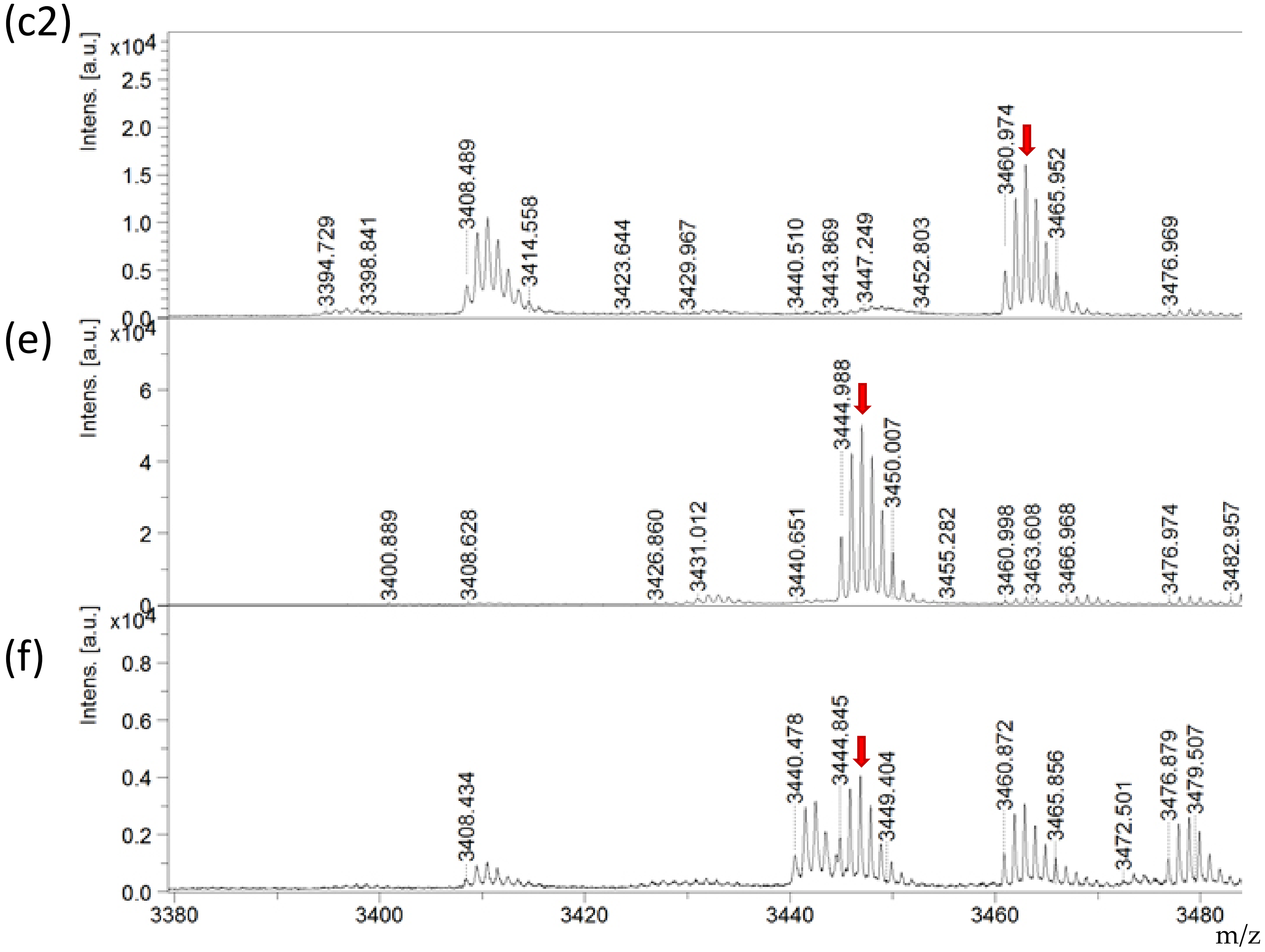

Supplementary Figure S2. MALDI-TOF-MS spectra of purified bacteriocins produced by *Leuconostoc mesenteroides* 406 and 213M0. Samples: peak-I of 406 (a), peak-I of 213M0 (b), peak-III of 406 (c1, c2), peak-III of 213M0 (d), peak-IV of 406 (e), peak-IV of 213M0 (f), peak-II of 213M0 (g), and blank (h). Red arrows indicate the highest peak in each analysis.

(g)

(h)

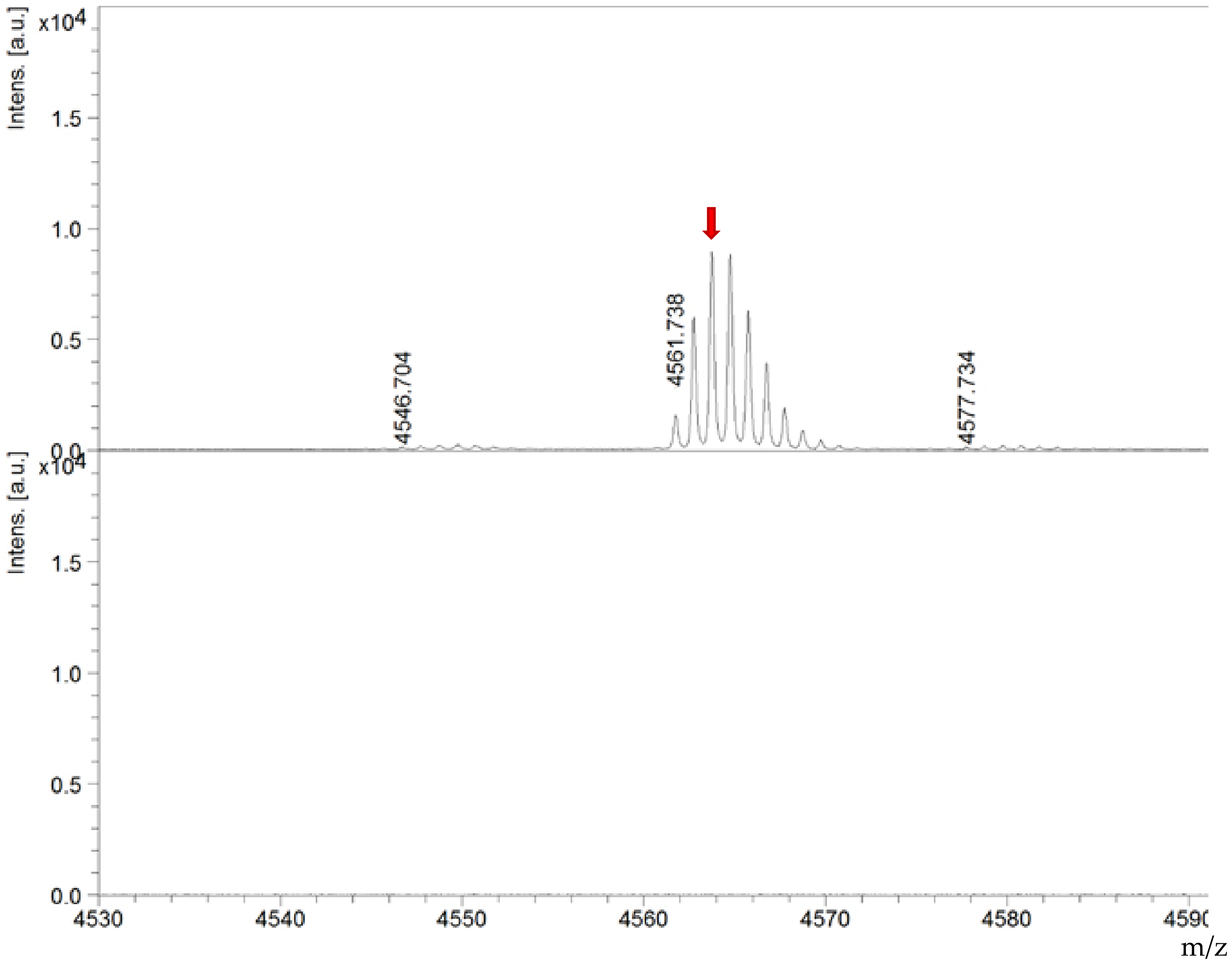

Supplementary Figure S2. MALDI-TOF-MS spectra of purified bacteriocins produced by *Leuconostoc mesenteroides* 406 and 213M0. Samples: peak-I of 406 (a), peak-I of 213M0 (b), peak-III of 406 (c1, c2), peak-III of 213M0 (d), peak-IV of 406 (e), peak-IV of 213M0 (f), peak-II of 213M0 (g), and blank (h). Red arrows indicate the highest peak in each analysis.

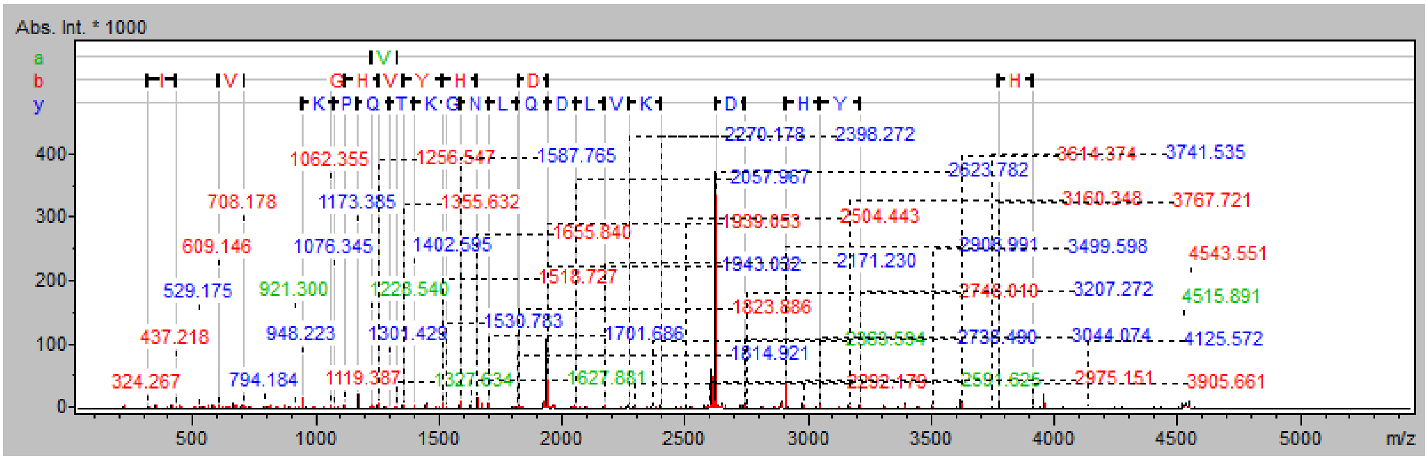

Supplementary Figure S3. MS/MS spectra of mesentericin M.

Supplementary Table S2. Monoisotopic mass (m/z) of fragment *a*-, *b*-, and *y*-ions in MS/MS spectra of mesentericin M. (precursor $[M+H]^+$ = 4561.7384)

#	a	a*	b	b*	Seq.	y	y*	#
1	110.0713		138.0662		H			41
2	296.1506		***324.1455***		W	4424.2575	4407.2309	40
3	409.2347		***437.2296***		I	4238.1782	4221.1516	39
4	466.2561		494.2510		G	***4125.0941***	4108.0675	38
5	581.2831		609.2780		D	4068.0726	4051.0461	37
6	680.3515		708.3464		V	3953.0457	3936.0191	36
7	793.4355		821.4305		L	3853.9773	3836.9507	35
8	850.4570		878.4519		G	***3740.8932***	3723.8667	34
9	921.4941		949.4890		A	3683.8717	3666.8452	33
10	1034.5782		***1062.5731***		I	3612.8346	3595.8081	32
11	1091.5996		***1119.5946***		G	***3499.7506***	3482.7240	31
12	1228.6586		***1256.6535***		H	3442.7291	3425.7026	30
13	1327.7270		***1355.7219***		V	3305.6702	3288.6436	29
14	1490.7903		***1518.7852***		Y	***3206.6018***	3189.5752	28
15	1627.8492		***1655.8441***		H	***3043.5384***	3026.5119	27
16	1724.9020		1752.8969		P	***2906.4795***	2889.4530	26
17	1795.9391		1823.9340		A	2809.4268	2792.4002	25
18	1910.9660		***1938.9609***		D	***2738.3897***	2721.3631	24
19	2008.0188		2036.0137		P	***2623.3627***	2606.3362	23
20	2136.0774	2119.0508	2164.0723	2147.0457	Q	2526.3100	2509.2834	22
21	2264.1723	2247.1458	***2292.1672***	2275.1407	K	***2398.2514***	2381.2248	21
22	2363.2407	2346.2142	2391.2357	2374.2091	V	***2270.1564***	2253.1299	20
23	2476.3248	2459.2983	2504.3197	2487.2932	L	***2171.0880***	2154.0614	19
24	2591.3518	2574.3252	2619.3467	2602.3201	D	***2058.0039***	2040.9774	18
25	2719.4103	2702.3838	2747.4052	2730.3787	Q	***1942.9770***	1925.9504	17
26	2832.4944	2815.4678	2860.4893	2843.4628	L	***1814.9184***	1797.8919	16
27	2946.5373	2929.5108	***2974.5322***	2957.5057	N	***1701.8343***	1684.8078	15
28	3003.5588	2986.5322	3031.5537	3014.5272	G	***1587.7914***	1570.7649	14
29	3131.6538	3114.6272	***3159.6487***	3142.6221	K	***1530.7700***	1513.7434	13
30	3232.7014	3215.6749	3260.6963	3243.6698	T	***1402.6750***	1385.6484	12
31	3360.7600	3343.7335	3388.7549	3371.7284	Q	1301.6273	1284.6008	11
32	3457.8128	3440.7862	3485.8077	3468.7811	P	***1173.5687***	1156.5422	10
33	3585.9077	3568.8812	***3613.9027***	3596.8761	K	1076.5160	1059.4894	9
34	3682.9605	3665.9339	3710.9554	3693.9289	P	***948.4210***	931.3945	8
35	3739.9820	3722.9554	***3767.9769***	3750.9503	G	851.3682	834.3417	7
36	3877.0409	3860.0143	***3905.0358***	3888.0092	H	794.3468	777.3202	6
37	4005.0995	3988.0729	4033.0944	4016.0678	Q	657.2879	640.2613	5
38	4168.1628	4151.1362	4196.1577	4179.1311	Y	529.2293		4
39	4255.1948	4238.1683	4283.1897	4266.1632	S	366.1660		3
40	4352.2476	4335.2210	4380.2425	4363.2159	P	279.1339		2
41					Y	182.0812		1

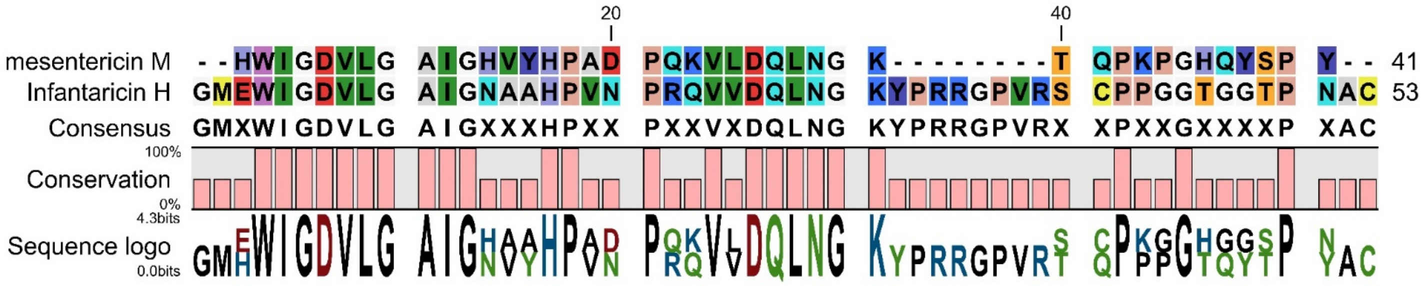

Supplementary Figure S4. Sequence alignment of mesentericin M and infantaricin H.
